# A collective case study of the features of impactful dementia training for care home staff

**DOI:** 10.1186/s12877-019-1186-z

**Published:** 2019-06-25

**Authors:** Claire A. Surr, Cara Sass, Michelle Drury, Natasha Burnley, Alison Dennison, Sarah Burden, Jan Oyebode

**Affiliations:** 1 Centre for Dementia Research, School of Health and Community Studies, Leeds Beckett University, Leeds, LS1 3HE UK; 2 Centre for Applied Dementia Studies, University of Bradford, Bradford, UK

**Keywords:** Care homes, Dementia, Education, Long-term care, Staff training, Workforce development

## Abstract

**Background:**

Up to 80% of care home residents have dementia. Ensuring this workforce is appropriately trained is of international concern. Research indicates variable impact of training on a range of resident and staff outcomes. Little is still known about the most effective approaches to the design, delivery and implementation of dementia training. This study aimed to investigate the features and contextual factors associated with an effective approach to care home staff training on dementia.

**Methods:**

An embedded, collective case study was undertaken in three care home provider organisations who had responded to a national training audit. Data collected included individual or small group interviews with training leads, facilitators, staff attending training, managers, residents and their relatives. Observations of care practice were undertaken using Dementia Care Mapping. Training delivery was observed and training materials audited. A within case analysis of each site, followed by cross case analysis using convergence coding was undertaken.

**Results:**

All sites provided bespoke, tailored training, delivered largely using face-to-face, interactive methods, which staff and managers indicated were valuable and effective. Self-study booklets and on-line learning where were used, were poorly completed and disliked by staff. Training was said to improve empathy, knowledge about the lived experience of dementia and the importance of considering and meeting individual needs. Opportunities to continually reflect on learning and support to implement training in practice were valued and felt to be an essential component of good training. Practice developments as a result of training included improved communication, increased activity, less task-focussed care and increased resident well-being. However, observations indicated positive well-being and engagement was not a consistent experience across all residents in all sites. Barriers to training attendance and implementation were staff time, lack of dedicated training space and challenges in gaining feedback on training and its impact. Facilitators included a supportive organisational ethos and skilled training facilitation.

**Conclusions:**

Effective training is tailored to learners’, delivered face-to-face by an experienced facilitator, is interactive and is embedded within a supportive organisational culture/ethos. Further research is needed on the practical aspects of sustainable and impactful dementia training delivery and implementation in care home settings.

**Electronic supplementary material:**

The online version of this article (10.1186/s12877-019-1186-z) contains supplementary material, which is available to authorized users.

## Background

Care homes provide care to 19–38% of people with dementia in Western countries [1, 2] and up to 80% of people living in care homes are thought to have dementia [2, 3]. In order to be able to deliver high quality person-centred care for this group, care home staff need to be provided with appropriate training that supports them to have the right knowledge, skills and attitudes [4, 5]. In England, there have been a range of initiatives, led by government over the last ten years to ensure the health and social care workforce receives appropriate dementia training [6–11]. However, in addition to ensuring the availability of training, there is a need to ensure that training is of high quality to provide the best chance of effecting practice change. A number of systematic reviews have examined research on the effectiveness of dementia training for the care home workforce in relation to a range of outcomes including the general benefits of training [12], impact on resident functional ability and quality of life [13], improving staff communication skills [14] and for supporting complex resident behaviours [15, 16]. The studies report variable impact of staff training on these outcomes. Training appears to most consistently support improvement of general care home staff skills [12], communication [14] and support for residents in activities of daily living [13]. However, there are inconsistent findings in relation to the impact of training programmes on resident outcomes such as behaviours (e.g. agitation, anxiety, neuropsychiatric symptoms) [13–16] and quality of life [13]. The reviews generally conclude that there is limited robust evidence for training efficacy due to methodological weaknesses in study designs and lack of follow-up over time. Where studies have included longer follow-up any positive results observed are generally not sustained. Few reviews consider features of effective training. One systematic review examining the challenges to and strategies for implementation of training in practice [5] identified the key challenges to include low staff attendance, lack of organizational support, and financial limitations. Therefore, there is limited available evidence on the most effective approaches to the design, delivery and implementation of impactful dementia training in care home settings.

The *What Works in dementia education and training? (What Works?)* study aimed to investigate the elements of an effective approach to dementia training and education for the health and social care workforce. This was achieved through conducting: 1) a systematic literature review of current evidence (see [17]); 2) a national audit of health and social care providers, commissioners and training providers on currently available dementia training; 3) a survey of staff who had completed programmes reported in the audit to assess their dementia knowledge, attitudes and confidence; 4) multiple case studies [18] in health and social care settings (general hospitals *n* = 3, mental health/community services *n* = 3, social care n = 3, general practitioner practices *n* = 1) who responded to the audit and whose training met good practice criteria identified from the literature review. In order to ensure enough data could be collected at each site to provide an in-depth picture [19], we aimed to recruit three case study sites from each setting type. This was deemed feasible within the project resources and timescales but was sufficiently large to permit cross-case comparison.

The study was underpinned by two theoretical models for the evaluation of training. Richards and DeVries’ [20] Conceptual Model for Dynamic Evaluation of Learning Activities, explores training *design* and *facilitation* processes. Kirkpatrick’s [21, 22] four-level model for evaluation of training interventions examines 1) learner reaction to training, 2) extent of learning in terms of knowledge, attitudes and confidence, 3) staff behaviour change, and 4) practice results or outcomes.

This paper reports a collective case study of the three social care case studies, which were all undertaken in care home settings.

## Aims

The case studies aimed to understand the features and contextual factors associated with good practice regarding the design, delivery and implementation of dementia education and training and its impact on care practices.

The research questions addressed were:

1. What models of dementia education and training were sites adopting?
2. How did staff perceive the training?
3. How did the training impact on staff knowledge, attitudes and practices?
4. How did people with dementia and their family members experience care in homes/units where staff had received training?
5. What were the specific barriers and facilitators to effective training implementation?

## Methods

We employed an embedded [23], collective [19] case study design.

### Case selection

A ‘case’ was defined as a care home provider organisation, which could include a single care home or multiple sites, as long as staff at all sites accessed the same training programmes. Eighteen social care providers in England and Scotland, including fourteen care home providers and four domiciliary care organisations who had responded to the audit were considered for inclusion. They were shortlisted using a positive deviance approach [24] by researchers blinded to site identity, and then ranked against a set of good practice criteria. These criteria were developed from the outcomes of the literature review [17]. They included how comprehensively training covered subjects and associated learning outcomes within the national Dementia Training Standards Framework for England [25] alongside training length and delivery methods (see Additional file 1 for full criteria and shortlisting process).

We had aimed to include at least one domiciliary care site in the three case studies. However, neither of the two sites which achieved high ratings against the good practice criteria were able to participate due to staffing issues affecting key individuals who would have needed to support the research. The three top ranking care home sites that were approached all consented to participate.

### Data collection

Consistent with a multiple case study approach [18], a range of data types were collected at each site (see Table 1) including semi-structured interviews with the dementia training lead, training facilitators and home managers and semi-structured individual or focus group interviews with staff who had attended training. Interviews were facilitated using a topic guide but conducted flexibly by the researcher to gain a thorough understanding of individuals’ experiences and views. Topic guides were unique for each participant type e.g. managers, training leads, training facilitators, staff, but contained questions based around the Richards and DeVries and Kirkpatrick Frameworks including organisational culture and processes (e.g. Could you tell me a bit about your organisation’s training strategy and the place of dementia training within this?), training design and delivery (e.g. What aspects have gone well in organisation and delivery and what has proved more tricky?), reactions (e.g. You’ve all taken part in [insert description] dementia training recently. Could I ask your opinions on the training you received?), learning and behaviour (e.g. Thinking about those team members who received [insert name of training here], can you identify any changes in their knowledge, or their competency in relation to dementia?) and outcomes (e.g. Do you think the training programme is having the impact you hoped for on care? Can you give us some examples?). They were audio recorded and transcribed verbatim, with interviews lasting for 30–60 min and focus group discussions around 60-min. The focus group discussions used the same topic guide but also included vignettes that presented a short story of the experiences of a person living with dementia in a care home in written and pictorial format. Focus group participants were asked to identify examples of good and poor practice contained within the vignettes, which helped to explore their knowledge and attitudes towards dementia care. The vignettes were developed by members of the project’s expert by experience group, which was comprised of people living with dementia and their family members.

**Table 1:** Summary of data collected and the research questions it addressed

Data collection method	Participant/collection focus	Research question addressed
Semi-structured interviews	Dementia training lead Staff who facilitated the training Home manager Care home residents and/or their relatives	1, 2, 3, 5 1, 2, 3 3, 5 4
Individual, small/focus group interviews (2–6 members)	Staff who had attended training	2, 3, 5
Observations	Training delivery Care practice	1, 2, 3 3, 4, 5
Audit	Training materials	1
Satisfaction cards	Care home residents with dementia and/or their family members	4

Each site provided copies of the training materials, which were audited using a good practice in training tool developed by the research team [26], based on the findings of the systematic review [17]. This includes items such as content and how well it mapped to the Dementia Training Standards Framework, whether it used interactive delivery methods, accuracy and readability of materials, tailoring to audience and training length. Researchers observed training sessions being delivered to staff, recording data using a qualitative observational template developed by the study team, based on the underpinning theoretical models. Short satisfaction cards, including three fixed (How satisfied are you with this service? How well did the staff understand your feelings and needs? How well were staff able to answer your questions about dementia?) and one open-response question (Any other comments about your care either positive or negative?), were given to care home residents with dementia and/or relatives. Respondents were also invited to take part in a telephone or face-to-face interview to discuss their care experiences. Only one resident in one of the sites completed an interview.

Care was observed in at least one unit of each participating site using Dementia Care Mapping (DCM) [27]. DCM collects data on residents’ experiences of care including behaviour (from 23 possible codes; Behaviour Category Code – BCC), level of mood and engagement (from a six-point scale (− 5, − 3, − 1, + 1, + 3, + 5: Mood and Engagement Value – ME)) and the quality of staff interactions with residents (Personal Enhancers and Personal Detractors). Up to eight hours of observation over both morning and afternoon periods were conducted by study researchers trained in DCM in public areas of the care home. As dementia training had been provided in all case study sites for a number of years prior to the study and was ongoing during data collection, no data was able to be collected before dementia training commenced. Therefore, analysis focussed on whether the outcomes the training aimed to achieve e.g. person-centred care, skilled communication, resident well-being, were present in the care homes.

### Consent and ethical issues

Ethical approval for the study was given by the Yorkshire and the Humber – Bradford Leeds NHS Research Ethics Committee [REC Ref 15/YH/0488]. The research team made the initial approach to participate to the individual who completed the audit earlier in the project, and arranged to visit the care home to meet with key staff such as the owner, training lead, facilitators and unit managers. Once formal written organisational consent from senior management was gained, the researcher visited each site again and gained written informed consent from all study participants. Where a resident lacked capacity to give informed consent, advice on their participation was gained from a relative or staff consultee in accordance with Mental Capacity Act [28] guidance. Adopting consent processes utilised in previous studies that have included general observations of care practices with people with dementia [29], verbal approval to record anonymised data was gained from residents and staff prior to DCM observation. In keeping with the principles of process consent [30] researchers assessed ongoing consent throughout. To ensure all individuals within the care home were aware of ongoing observations posters were displayed in prominent positions on the units before and during observation period, containing a photograph of the researcher and giving details about the study and how and with whom to ask questions or raise a concern.

### Data analysis

The study team undertook analysis of the full set of data for each case study site individually followed by cross-case analysis. Interview, focus group and training observation data were analysed using the thematic analysis method, template analysis [31, 32] using NVivo 11 [33]. Starting with a priori themes drawn from the underpinning theoretical frameworks [20, 22] a coding template was developed that underpinned data analysis across the whole study. This was achieved through CAS, JO, CS, MD, SB and NB undertaking collaborative coding of three initial transcripts (one social care, one acute care and one mental health Trust) and discussion of the identified themes. A further six transcripts (representing the range of service settings) were then coded by CS, MD and NB to refine the template. This final template was then used to code the remaining data.

DCM data were analysed using standard DCM guidelines, including preparing summaries of data at an individual resident and group level. Copies of training materials were reviewed and their content mapped against the learning outcomes contained within the Dementia Core Skills Education and Training Framework [25]. The audit tool [26] of good practice in dementia training was used to audit each training programme. The responses to patient and carer satisfaction cards were summarised using descriptive statistics and manual thematic analysis.

Once analysis of each data source for a site was complete, a within case analysis [19] was conducted. This involved summarising each data source, triangulating across sources, and synthesising into a written ‘story of the case’ [34]. This was followed by cross-case analysis [19] across the three sites using convergence coding [35]. Convergence coding involved creation of a data grid highlighting themes and findings, supporting comparison of areas of agreement, partial agreement and dissonance [36].

## Results

The organisations recruited varied in terms of size and number of units participating in the study (Table 2), although all were within provider organisations who owned a small number of care homes (≤7) and were located across England and Scotland. All had an internal training lead/trainer who was responsible for delivery of dementia training across all homes within the organisation. The key themes and issues identified in the analysis are presented by site in Table 3.

**Table 2:** Characteristics of case study sites

Site	Site(s) data collected at	Key training staff	Training packages (what observed)	Training delivery methods	Staff participants	No of returned satisfaction cards resident/family interviews conducted	DCM data collected
SC 040	Two care homes within the group’s portfolio of 7	Internal training lead/facilitator	1) Dementia e-learning (within induction) 2) Introduction to dementia (3.5 h) (full session) 3) Dementia Skilled (6-month, self-directed course developed by Scottish Social Services Council and NHS Scotland) + monthly tutorials) (1 monthly tutorial)	Didactic content, PowerPoint slides, discussion	1 Training Lead 1 Training facilitator 3 Unit Managers 10 staff who had attended training	10 cards 1 interview with resident with dementia	Unit A 4 h on single day, 5 participants Unit B 6 h over 2 consecutive days, 7 participants
SC042	Three of the organisation’s specialised units for people with dementia within the groups portfolio of 7 homes	Training lead Dementia Lead	1) Induction programme: dementia related content (1-2 h) (dementia components) 2) Dementia awareness for care staff (2 h) and for non-clinical staff (2 h) (full session) 3) ‘Behaviours that challenge’ (2 h) 4) Meaningful activities (2 h) 5) Dementia Training Programme (specialist – self-directed)	Didactic content, flip chart/white board, discussion, handouts, interactive exercises	1 Training Lead 1 Dementia Lead 2 Unit Managers 11 staff who had attended training	24 cards	Unit C 6 h over 2 consecutive days, 9 participants Unit D 5 h over 2 consecutive days, 5 participants
SC 076	Single care home within the groups portfolio of 4	Training lead (also group Director) Training facilitator	1) Dementia awareness (4 h) 2) Person-centred dementia practice (4 h) (full session)	Didactic content, discussion, pair’s exercises, case scenarios, video clips, written exercises and other interactive activities.	1 Training lead 1 Training facilitator 1 Unit Manager 12 staff	8 cards	Unit E, 9 h over 2 consecutive days, 6 participants

**Table 3:** Summary of key findings and themes across case study sites

Major theme	SC040	SC042	SC076
	Sub-themes		
Training design and delivery	Introductory programme developed by training lead and tailored to care provider staff but not to organization. Didactic Too much content for allocated time Generic national standard training workbook. Format adapted by training lead to include face-to-face monthly sessions with reflective exercises. Gaining staff feedback challenging	Wide range of training available at different levels and for different staff groups Designed by training lead who had considered learner needs Majority delivered using small group discursive sessions One programme delivered by self-directed work book	Designed by experienced internal training facilitator Bespoke training tailored to organization and staff attending Combination of minimal didactic PowerPoint based content and interactive group discussion, exercises and case scenarios Minimal use of written materials and use of video-based scenarios Training delivered in care home lounge, not enough seating for all staff who had to sit on floor Rushed pace at times
Staff reactions	Generally positive Relevant to role and own practice Valued face-to-face delivery and regular two-hour sessions spread over a prolonged period Valued case studies	Training felt to be generic and not easily transferable to considering how to work with individual residents Felt to be too basic/to cover content they already know by some staff Valued small group, face-to-face learning and interactive learning methods Including staff from range of roles in training seen as positive Simulation training evoked strong emotional and empathic reactions Disliked self-directed learning via work books	Preference for ‘hands on’ interactive methods Positive response to video-based scenarios as helping to understand what it might be like to live with dementia Importance of safe and relaxed environment to support discussion and asking questions External training also valued and seen as impactful
Learning	Level of training good for someone with limited prior experience, but provided limited new learning for more experienced staff. Understanding of lived experience of dementia Understanding individual needs and differences Empowerment to challenge poor practice Generally positive attitudes, but a few who appeared not to do so	Evidence of knowledge gains on range of topics Improved understanding of dementia and changed attitudes towards those with it e.g. more patience Understanding of lived experience of dementia Practical skills developed e.g. writing care plans Learning through study work books hampered understanding Learning from each other in and outside of classroom Ideas for new approaches and practices Generally positive attitudes, but few staff who on occasions appeared unsure how to support more complex needs	Simulation and experiential learning helped develop empathy Many staff reported having a more person-centred understanding of people’s individual needs Some staff reported being unsure about what learning had been achieved given their existing experience Observations showed staff had a positive attitude and knowledge of the need for activity, occupation and engagement
Behaviour	Understanding, interpreting and reacting differently to resident behaviours Improved communication Introducing new activities Shift from task focused to person-centred care Offering more choice to residents Not always clear changes due to training Staff interactions mainly positive and skilled, but occasions on one unit when practices were less person-centred.	More empathic care approaches Better able to diffuse difficult care situations Development of enriched care plans and delivery of care that is more individualized Introducing memory boxes and meaningful activities Staff interactions mainly positive and skilled, but occasions on one unit when practices were less person-centred.	Difficult for training lead to assess whether staff are implementing in practice Improved communication Provision of personalised activities Providing care at the right pace Changing the environment and care procedures Improved support for relatives Overall sensitive care that supported engagement, with few occasions where resident choice was limited.
Experiences of care	Improved resident emotional and physical well-being Different experiences on each unit where observations carried out, with one offering residents greater opportunities for activity and generally higher well-being. Satisfaction of residents and relatives generally high although some suggestions offered for way care could be improved.	Staff perceptions of improved resident well-being due to increased activity and engagement More positive staff: resident relationships Limited evidence of resident activity and engagement during care practice observations Residents' generally experiencing neutral to positive mood. Satisfaction of residents and relatives high	Staff perceptions of increased resident well-being and reduced distress Evidence of good range of activity and engagement tailored to individual residents. Some residents had less opportunity to engage than others. Mood and engagement levels were on average above neutral trending towards positive Satisfaction and residents and relatives high
Barriers to training implementation	Staff expected to complete training in their own time E-learning not viewed positively by staff Difficulties releasing staff to attend training Lack of appropriate training facilities Difficulties evaluating impact of training Lack of staff motivation to put learning into practice Staffing levels and turnover	Low status profession Tensions between staff and relatives due to conflicting views about care Staff turnover Use of self-directed learning Expectations of completing training in own time Difficulties releasing staff to attend training Lack of time to put training into practice Embedding changes sustainably	High costs of external training Just accessing internal training can create ‘inward looking’ Demands of managing training leadership alongside another role within the organisation Financial constraints in being able to access technology to support interactive learning Lack of time and staff shortages Lack of formal curriculum and quality assurance for social care sector Single trainer who is not linked to a community for peer support Gaps in facilitator knowledge Trying to meet learning needs of clinical and non-clinical staff in mixed-group training Staff wariness of and confidence using technology Lack of dedicated training space Challenges in getting feedback about training from staff
Facilitators of training implementation	Organisational culture that valued training Training lead spent time on care home units Management support for staff Adapting standard training to make it accessible Being in a small organisation that could listen to staff and offer training flexibility Skilled facilitator Small, mixed-role and unit training Delivery methods that made training memorable, linked theory to practice and encouraged reflection Incentives to complete training (badge) Peer support and team-working Committed and motivated staff	Dedicated training room Mixed role training sessions Flexible and committed training lead Having a practice and training facilitation experienced dementia lead and training lead Supportive management Strong leadership for dementia training Proactivity by training lead in accessing additional resources	Good organisational support Ability to access external training Having an dedicated internal trainer Staff undertaking training during paid working hours Facilitator skill Motivated and proactive staff Engaged unit managers Using supervision to reinforce and feedback on training implementation Peer support

### Design and delivery

All sites offered a range of training provision (Table 2) that was mostly bespoke and developed by the training lead. The majority of training was delivered face-to-face in small groups, with some sites including other delivery methods. In one site, a standardised workbook that covered required dementia training content for Scotland was used. However, the training lead had tailored the delivery method by including additional monthly face-to-face discussion groups where staff could reflect on application of learning, recognising the importance of co-learning.

*We thought in order to change practice that it has to be facilitated within the team … all the reflective exercises are about people that they actually care for. Thought it was more real … and group facilitation rather than just giving people the folder with the information. (Training Lead SC040)*

In another site, a self-directed workbook was also used but the approach was under review due to both the local Council and the training lead identifying this method was not appropriate, as the training was not being completed.

*They are given a booklet but basically left with it*. (Dementia Lead SC042)

The training facilitator in one site highlighted how she had removed as much written material as possible from the training, upon recognising that staff did not find it helpful to their learning.

*Giving lots of hand-outs was not effective because it was just people getting stressed out because they couldn’t find a hand-out or they had too much information to read to process and they weren’t really focussing on the training (SC076 Training Facilitator)*

Dementia training was offered to all staff working in the care homes irrespective of role.

*You’re not going to have laundry staff that are experts in dementia because they don’t have to be. It’s not their role. But you still want your workforce to be fit for purpose and have an awareness with the client group they’re working with. (Training Lead SC040)*

During training observations it was noted that the training leads in each site delivered content flexibly to meet the needs of the group, for example by tailoring examples they provided to the group participants and their role and asking for and responding to learner’s own practice examples to inform discussion. The trainers recognised the importance of tailoring provision to the needs of the organisation and range of staff attending.

### Reaction to training

Staff responses to the training were generally positive across the three sites. During focus groups, interviews and immediately following training staff made comments such as *interesting* (SC040 Staff Member 026), *informative* (SC040 Staff Member 025) and t*he best training I’ve ever been on* (SC042 training observation field note). Key themes related to training reaction included the value of small group, face-to-face learning, a dislike for e-learning and the benefits of using case scenarios.

Overwhelmingly staff identified the importance of face-to-face learning and the ongoing support provided by the sites for staff during and after training.

*I find personally I understand things better when it’s in a training setting, er, there is a group of you, when you know, er, giving ideas and all talking together about it rather than a question on a page. (SC042 Staff Member 034)*

In one of the two sites (SC040) that utilised self-directed study via a work book, the training lead had added monthly reflective face-to-face sessions. However, one staff member commented that they would prefer it to be delivered as a full face-to-face session rather than

*… having people go home and work on it on their own and then come back into the course just to talk about it. (Staff Member SC040 013)*

In the other site the delivery approach had not yet been revised and staff commented on how unhelpful they found the method.

*because it is how you respond to a person verbally. You can’t do that out of a book can you?* (SC042 Focus Group P1)

On-line modules formed a component of induction in one site and had previously been part of training in another, however this was not viewed favourably by those in leadership positions, who saw it as little more than a tick-box exercise.

*You know a monkey could sit and do it. (Unit Manager SC040 020). … ‘cause they can copy and they can say just tick tick, tick, that’s fine (SC076 Training Lead).*

Staff also noted they found interactive learning activities and the use of video or other forms of case study scenarios particularly helpful in helping them to apply learning to practice.

*Mostly the scenarios* … . *This scenario thing and it was exactly like, exact same as one of the residents in here. (SC040 Staff Member 013).*

*Videos have worked well … If you could find a decent video that supports a point that you’re trying to make and you can see it in practice it’s really good because issues that we have … role play is wonderful but it doesn’t really…it’s not an accurate simulation of someone with dementia. (SC076 Training Facilitator)*

### Learning

There was evidence from the interviews, focus groups (including vignette-based discussions) and observations of care practice that a range of learning had taken place. Key themes were gaining empathy and knowledge about the lived experience of dementia, and understanding individual needs. These themes were a consistent outcome of training across all three sites.

*I feel I’ve gained a lot of understanding about dementia and how it progresses and you’ve sort of put yourself in their shoes and you think well that could be me some day, so I would hope that whoever’s looking after me would give me the care that I would expect and understand. (SC040 Focus Group P4)*

*… you just feel as though you need to help them more, whereas before I’d have dismissed them. I won’t say I was awful but I would have, I would have thought: Oh silly old fool or … . Whereas now I think I’ve got much more empathy with them and feeling more towards them. (SC042 Focus Group P1)*

The importance of understanding and providing care that was person-centred and met individual residents’ needs was identified as a learning point by staff at two sites.

*Staff can step back and say ‘that’s why that person does that. Now we know what to do’. (Staff Member SC040 014)*

*So you’ve got to individualise when you’re caring. (SC076 Focus Group 3 P2)*

One staff member reported finding some content during the session overwhelming and that s/he only took in the information upon,

*… reflect[ing] on it when you’re on the floor. (SC040 Staff member 026).*

The learning that took place ‘on the job’ was also identified as important by a staff member at another site.

*I think for training is good in some ways but to be here is more life, true, real-life, the way it is. For me it can be both but to be here you learn more. (SC042 Focus Group, P2).*

Spreading training over 2-h sessions over a number of weeks, with some reflective activities to complete outside of the training room was also identified as helpful in supporting learning.

*[It gives me a chance to] go home and it’s good just to sit, relaxing, writing your scenarios. You know what you’ve to do and what you’ve to say and you get time to think about it. (Staff Member SC040 026)*

In another site opportunity to continue reflecting in a supported way outside of formal training was also offered through ‘drop-in’ sessions or provision of additional support materials.

*They’ve got you in the back of their minds on you, on their radar to help you with other stuff as well as the Booklet. (SC042 Staff Member 033).*

While most staff commented positively about the value of training, some of the more experienced staff in two of the sites indicated that for them there had been little new information covered in training they had attended.

*With the Induction Training, there was nothing, nothing added to what I already knew. (SC042 Staff Member 034).*

Whilst for other less experienced staff coverage of dementia in the initial induction was not in-depth enough to help them feel confident when commencing work in the home, or training content did not provide enough support to help them in the range of often challenging situations they might find themselves.

*… how to get out of situations if somebody has got hold of my hair, how do I get out of that? (SC042 Staff Member 033).*

### Behaviour change

Themes related to behaviour change included adopting a more empathic and understanding approach, improved communication, provision of meaningful activity, a shift from task to person-focussed care.

Staff in two of the care homes (SC040, SC042) identified how training had helped them to deliver care that was more empathic and was understanding of resident behaviours and what they communicated about individual needs.

*SC042 Staff Member P2: We’ve got one lady who goes back to when she was in the War and she was deported and she gets terribly upset and she thinks we’re keeping her in. So we just take her outside on the decking for a little bit, then she is okay. She’s not a prisoner of war anymore. ‘Cause she thinks we’re keeping her a prisoner. But I wouldn’t have known to treat her like that unless I’d known that that’s how dementia can affect you.*

*I: What might you have done before?*

*P2: Well, probably said, ‘Look you’re okay, sit down, have a cup of tea’ and basically get on with it, which I probably would have.*

As a result of improved staff understanding one manager noted there was a demonstrable reduction in drugs used to manage behaviour in people with dementia, due to staff being able to support needs through psychosocial approaches.

*There has been a real marked reduction in the number of drugs and that I can prove. That’s documented and it’s easy to do. (Unit Manager Sc040 020)*

In two sites (SC040, SC076) improved staff communication was a behavioural outcome of training. Staff gave examples of approaches the training had taught them, such as wording questions so residents can give a yes/no answer. Keeping language simple and using picture prompts. There was also increased confidence in staff to communicate with residents.

*I’m having a joke with them you know, talk about their families and they like talking about- you know talking about their families.. (Staff Member SC040 026).*

*Talk softer, come down to their level. It’s easier just to say ‘here’s your dinner’ you know and put it in front of them. I don’t do that anymore (SC076 Focus Group 1 P1)*

**Fig. 1:**
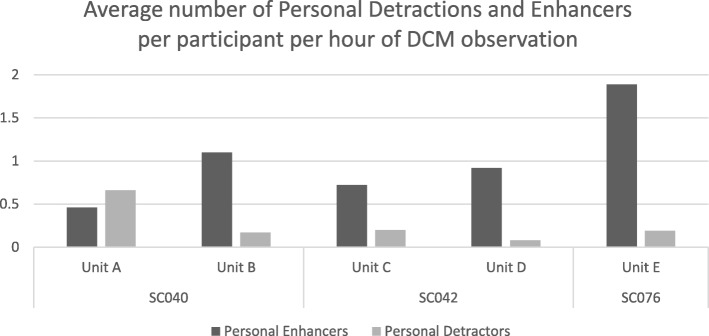
Average number of personal detractors and enhancers observed per participant per hour by site and unit

The DCM data showed that in four of the five units observed there were more personal enhancers than detractors observed on average, per participant than detractors (see Fig. 1) and overall detraction levels were low. In one unit (B) at site SC040, however, more detractors were observed than enhancers during the mapping period. This indicates that in that unit on the days observations took place not all staff were communicating in person-centred ways.

All three sites indicated that implementing new activities in the home had resulted from staff attending training. In one home (SC040) this included one-to-one engagement, hand massages and cookery classes. They had also arranged visits from external professionals who gave Indian Head Massages, ran dance classes or delivered group music sessions. The latter two were particularly highlighted as being enjoyed by the residents.

*You would not believe how good it is [the music session], it’s just amazing, such a good feeling. (Unit Manager SC040 020)*

*They just get on with it, some of them make themselves a drink and stuff. And I think just not saying: ‘Oh you can’t do that’ is wrong. It’s about observing them doing it, making sure they’re safe. I think that’s a good thing we’ve learnt from training, let them be independent. (SC042 Staff Member 802).*

In site SC076 staff used a new SMART TV to look for old films, singers or YouTube clips that residents might enjoy. In site SC040 the maintenance worker had started promoting vegetable-growing amongst the residents after attending training. He understood what the residents needed in order to support them to take part in the project. The residents were able to sow the seeds, care for the potatoes, harvest them and then peel them ready to be eaten.

Making a shift from a task focussed to person-centred care was another behaviour change reported. In site SC040 staff commented that they felt they had ‘permission’ to focus on person-centred care such as activities and spending time with residents, rather than feeling they should be completing tasks. This change in behaviour was noted by the training lead.

*[They are no longer focussed on] they have to do this for this time and this for this time and the individual gets lost so I think we’re breaking that down. (Training Lead SC040)*

In site SC076 the manager identified that person-centred approaches had also been extended to the support of family members.

*I think people exhibit more patience, more individualised care, more person-centred care. I think that goes for relatives as well. We support relatives in an individualised person-centred way, because some of the relatives need that care (Home Manager SC076)*

Staff in one care home noted how training was one part of the bigger picture that had supported a shift in culture.

*It validated that for us we were on the right track. Obviously things always need to be tweaked, I know that, but I think it was giving a bit of confidence that we’re on the right track. (SC040 Focus Group P3).*

### Outcomes and impact

Themes related to outcomes and impact included improved resident well-being and decreased distress; disparities and variability of experience; and high resident and relative satisfaction.

Staff across all three sites consistently stated they felt that, as a result of the changes staff had made to practice, residents were experiencing greater well-being and were less frequently distressed.

*I do think the training has impacted on their wellbeing in a positive way [. . . ] The carers take a more, a better interest in, you know, what the person like(s) and needs are and how they can make it a better day for them. (SC040 Staff Member 014)*

*It made them less agitated, they had something to concentrate on, something to do which improved their mood massively. When you work out what activity is right for the right person you then get a better mood all day. (Home Manager SC042)*

**Fig. 2:**
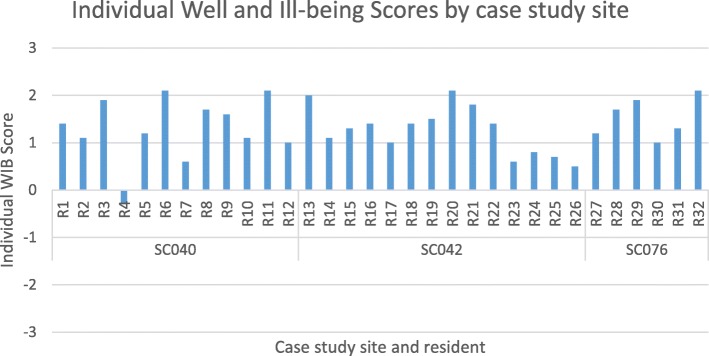
Individual Well and Ill-being Scores by setting

Our observations of care showed that while resident well-being was generally moderately good and levels of ill-being were low, this did differ between units within the same organisation and across different residents living in the same unit. Figure 2 presents the average Mood and Engagement Value per resident over the period they were observed, known in DCM as their Individual Well and Ill-being Score.

**Fig. 3:**
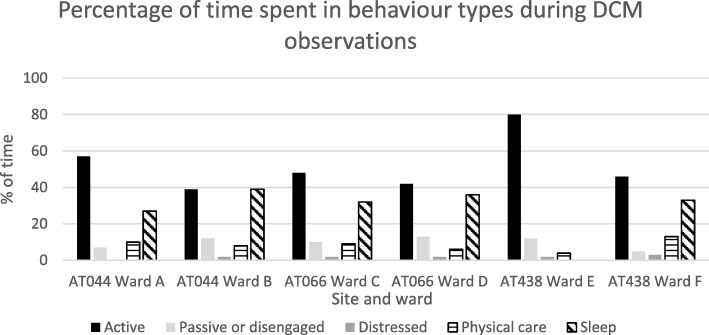
Percentage of time spend in different behaviours during DCM observations

We found similar results when looking at engagement in activities (see Fig. 3). In some units, residents spent more of the observation period in disengaged and distressed behaviours (e.g. passive observation, disengagement, sleep, distress and repetitive behaviours) and less time engaged in active behaviours (e.g. interacting with others, singing, reminiscing, physical exercise, sensory stimulation, work-like activity etc).

The residents’ and relatives’ satisfaction cards showed high overall satisfaction with care received and respondents felt staff understood their/the residents’ feelings and needs and were knowledgeable about dementia. The qualitative comments included positive aspects and some suggestions for ways care could be improved.

*We’re only allowed one shower a week. They have a nice way with them. (Respondent 3 SC040)*

*My mum used to live in another home but since she came here she is much happier. The dementia care staff know their stuff and nothing is too much trouble. (Respondent 1 SC042)*

*My Auntie is very well cared for and all her needs are met. All the carers are very patient with her. There is always someone who can answer any questions I may have (Respondent 1 SC076)*

In one site, a resident chose to take part in an interview. They said that they felt they were given choices at mealtimes through being given a menu with two different meal options to choose between and believed that staff members respected these choices.

#### Training barriers

Despite the sites being chosen for the positive aspects of their training, all still experienced a range of barriers to delivery and implementation. Common barriers across the sites included staff time, staffing levels and turnover, lack of dedicated training facilities and difficulties in gaining feedback from staff.

### Staff time, staffing levels and turnover

In all three sites a lack of time, staffing levels and turnover were a challenge to training delivery and implementation. This included difficulties being able to free up staff to attend training due to difficulties covering shifts, the need to constantly train new staff in the more basic levels of training due to turnover and a lack of time for staff to implement learning in practice.

*Eight people is an awful lot of people off the floor, you can’t, it is just impossible to do (SC040 Manager 019)*

*Turnover at the moment is really quite difficult to manage (SC042 Dementia Lead)*

Two sites had previously required staff to undertake learning in their own time either via face-to-face or self-directed means. This had not been successful in terms of staff reaction to training or completion rates. As one manager stated:

*You can’t just expect them to pitch up and not be paid (SC040 Manager 020)*

*P1 It’s not completed by any means. It was meant to be completed ages ago, P2 I’ve lost mine. (SC042 Focus Group)*

### Lack of dedicated training facilities

In two of the sites there were no dedicated training facilities available, meaning training was delivered in a lounge or other room in the care home that was often cramped and unsuitable.

*Venues are normally an issue because you normally get put into a lounge. A lounge doesn’t have a lot of space really. Sometimes the rooms are quite small and that limits the number of people you can have in the room and limits, you might wanna do – can’t really facilitate or there may not be sufficient wi-fi… (Training Facilitator SC076)*

### Difficulties in getting feedback on training

In two sites the training lead/facilitators mentioned difficulties they experienced in getting honest and practical feedback from staff about how useful the training had been as well as impact on care practice.

*It’s difficult to get out because they all say “We enjoy the training”. “Great, ok, what did you like?” You can ask it verbally or you……if you ask it verbally you get a better answer. If you ask them to write it down it doesn’t really come through…all of it. “Which bit was particularly useful for you?” “Yeah, well everything.” Ok. There’s not really real constructive to feed back in. (SC076 Training Facilitator)*

*I can’t say I’ve had fabulous feedback in terms of change (SC040 Training Facilitator)*

#### Facilitating factors

Common facilitators of training delivery and implementation across the good practice sites included commitment of the organisation and management, skilled training facilitation and strong peer and team support.

### Commitment of the organisation and management

The importance of organisational and managerial commitment to dementia training was a strong feature of all of the sites. This included an organisational culture and ethos that valued training, home or unit managers who supported training attendance and implementation in practice, and strong leadership for dementia training via a dementia and/or training lead.

*As a company [name] are really, really keen and up there to make sure the staff are fit for purpose, well trained and can deliver good care and they feel quite passionate about it I think (SC040 Training Facilitator)*

*So, it has to come from the top. You can have the best carers in the world, but it makes no difference if the people at the top don’t want to actually give people time to learn, (SC042 Dementia Lead)*

### Skilled training facilitation

Skilled and flexible training facilitation was mentioned as a facilitator in all sites. The trainers made learning memorable and managers commented that staff often talked about dementia training when back on the units afterwards.

*[The Training Lead] is quite flexible, she will come into the homes if the homes are struggling or short staff and she’s got people that need to do training. She’ll come round here rather than go out there. (SC042 Manager)*

### Strong peer and team support

Having a staff team who were motivated to learn, supportive of one another and who felt empowered to make suggestions for practice change was a facilitator at all three sites.

*[Name of colleague] is really good at raising stuff. Because she’s an admin worker, her perspective is different. And she will quite often say: ‘But, why can’t you? Why?’ and sometimes in an organisation, that is what you need- people that will challenge, because otherwise you end up with, you all do it that way, because you all do it, and that way can lead to stagnation, bad practice. (SC076 Unit Manager)*

## Discussion

The case studies identified a range of elements of good practice in relation to training design, delivery and implementation that are applicable not only to dementia training, but to broader training delivery within care home settings. As was reported by Beeber et al. [5] the design and delivery methods utilised were important and in the case studies particularly impacted on staff reactions to training and subsequent uptake. Findings across the three sites strongly support the use of face-to-face delivery, interactive and engaging teaching methods and the tailoring of training to the setting and staff roles of those attending. The preference for and benefits of face-to-face, interactive training in care home settings are reported in the international research literature see for example [37, 38]. This were also a common feature of training delivery preferences of staff in other settings (e.g. acute hospitals [39]) within the broader What Works study. However, implementation of such methods is pragmatically challenging in light of the staffing and resource barriers that were identified at all sites, as well as the broad range of subjects and learning outcomes that staff training must address in order to meet national standards [40, 41] (see for example [42, 43]). Staffing issues and having the resources to support staff to attend and implement training have been reported as challenging within social care workforce development and intervention research [44–47]. This suggests that care provider organisations and researchers should consider resource and staffing issues and how they will be addressed or accommodated, before embarking on new programmes of staff training in care home settings.

In the case study sites, an organisational ethos and culture of commitment to dementia training, which was evidenced throughout the management team, helped to overcome some of the resource issues. This, coupled with the presence of dedicated training staff to develop, facilitate and champion training, provided a positive context in which training could be carried out and implemented despite the challenges. The importance of both top-down and bottom-up approaches to changing care practice through educational programmes in care home settings has been reported in other research. This includes active executive and management involvement and the presence of individual(s) to ‘champion’ implementation [13, 38, 47]. Where managers are seen as ‘far removed’ this can be a barrier to training implementation [46]. The organisational culture was also reflected in the peer support, and staff engagement in training attendance and in subsequent implementation. Resistance to change among staff teams [48] and the impact that individuals who are ‘rigid’, ‘closed-minded’ or ‘indifferent’ can have on colleagues’ motivation is another potential barrier [46]. This indicates that in the design of training programmes, trainers and organisations should not only consider the content and delivery but also how to prepare and engage the organisation and individual staff members. Without a team and organisational culture that is largely supportive of training and its implementation, the many barriers that exist are likely to prevent optimal impact [49, 50].

It was disappointing that we were not able to recruit any domiciliary/home care organisations into the study. It is likely that some of the issues, barriers and facilitators may be similar to those experienced in care home settings due to the similarities there are in demographics and prior educational experience of both workforces. However, we would also anticipate domiciliary care providers and staff to experience a range of additional challenges associated with lone working, use of zero hours contracts [51] and a geographically spread workforce.

### Limitations

There are a number of limitations in this study. While the case studies were in-depth, we were only able to include the three top-performing audit respondents in ‘best practice’ case studies. Therefore, the sample is not representative of the typical or average care home. Given staff had already accessed a range of dementia training, it was not possible to understand the direct impact on outcomes of individual training packages included in the case study. The respondents to the satisfaction survey for residents with dementia and their family members may reflect participation bias. Residents and family members who are more satisfied may be more likely to respond than others. Relatives who are dissatisfied may be concerned about raising issues if given their loved one is still being cared for in the care home. It is difficult to draw any firm conclusions about the impact of training on staff practice and resident outcomes from the observational data.

## Conclusions and recommendations

Despite care homes being one of the most researched settings in terms of dementia training and its impact, relatively little is still known about how the emergent design and delivery features of effective training (e.g. face-to-face, tailored, flexible, interactive) can be implemented practically. Likewise, while an understanding of the ideal setting conditions for training and other psychosocial interventions is evolving, how these can be facilitated and sustained is still poorly understood or implemented. More research is still needed on the practical aspects of sustainable and impactful dementia training delivery and implementation in care home settings.

This study has added to our understanding of effective dementia education and training for care home staff. It suggests that training that is most likely to lead to positive outcomes across staff reactions, learning, behaviour change and outcomes for people with dementia has the following qualities. It:

- Is delivered face-to-face to a small group using interactive methods such as discussion, case studies and practical exercises and activities;
- Is tailored to the setting and role of staff attending and was inclusive of all staff working in direct care and non-care roles;
- Provides ongoing support outside of the training room for staff to reflect on learning and implement training;
- Includes methods that support staff to engage with the lived experience of people with dementia;
- Is delivered by an experienced training facilitator who is able to engage and work flexibly with staff;
- Is one component of achieving an organisational commitment to and culture of person-centred care;
- Is supported by the home owners and management team in terms of resource and development of an organisational culture that values learning.

## Additional file

Additional file 1: Inclusion criteria and steps for selection of the case study sites. (DOCX 62 kb)

## Data Availability

The datasets generated and/or analysed during the current study are not publicly available but are available from the corresponding author on reasonable request.

## Abbreviations

BCC: Behavioural Category Code
DCM: Dementia Care Mapping
ME: Mood and Engagement Value
