# Supplementary material for: A collective case study of the features of impactful dementia training for care home staff
Source: BMC Geriatr. 2019 Jun 25;19:175. doi: 10.1186/s12877-019-1186-z (PMC6593517; doi:10.1186/s12877-019-1186-z)
Supplement: Supplementary file 1 — Inclusion criteria and steps for selection of the case study sites. (DOCX 62 kb) [file 12877_2019_1186_MOESM1_ESM.docx]

## **Additional file 1: Inclusion criteria and steps for selection of the case study sites.**

| **Criteria from proposal** | **Steps to put into practice** |
| --- | --- |
| The training that is being offered must have already been delivered to at least one group of staff of the organization | This applies to all who have returned survey 1, so all will be included in the next step. |
| The organisation must be providing training that meets all the learning outcomes specified in the Dementia Core Skills and Knowledge Framework awareness level, or all the learning outcomes of at least one of the specific elements at in-depth or leadership levels (Skills for Health and Health Education England, 2015). | 1. Identify all sites which, on survey 1, report meeting >95% or 75-94% of the learning outcomes of at least one subject area at Tier 1, Tier 2 or Tier 3. 2. Produce a table of those sites, ensuring all are anonymised, and referred to by code only 3. Group these by type of organisation (acute, specialist secondary care, primary care, social care) 4. Within each organisation type, allocate a rank derived by counting ‘met learning outcomes’ (score 1 for >75% met and 2 for >95% met) at each Tier. |
| We will aim for variation, if possible, in respect of level of training | 1. Research team review rankings table and, exceptionally, agree any changes in ranking in order to ensure variation in level and range of modules (e.g. if one organisation has training that meets >75% of learning outcomes for 5 modules and so scores 5, it may be ranked to take precedence over an organisation that meets >95% of learning outcomes for 3 modules, depending on the spread and level of modules covered) 2. This will lead to a final ranking on the criteria about meeting learning outcomes. |
| We will focus, where possible, on sites with identified innovative training practice or positive deviance in terms of outcomes, as derived from WP2, survey 2 data as this will allow us to explore facilitators of good practice. | Using responses to survey 1, organisations’ modules will then be compared on several indicators of good practice identified from the literature review. Training:   1. Is facilitated in small or large groups 2. Involves group based discussion 3. Has sessions of 60 minutes or longer 4. Has a total programme length of 8 hours or longer 5. Does not include ONLY online learning   A score of 1 point per training programme will be allocated for each indicator met and a second ranking will be produced for these aspects of evidence-based training. |
|  | Rankings for these two criteria will be compared. Any in the top 5 of both ranks will comprise an ‘A’ shortlist. A sensible data driven means of grouping the others as ‘B’ or ‘C’ list will be derived in the light of seeing the data. |
|  | Friends and Family data and Safety Thermometer data will be located for the organisations and they will be allocated to 1 of 3 groups, high, medium and low using data derived categories. |
|  | CQC reports will be inspected, and organisations will be rated based on outstanding/good, requires improvement, inadequate |
|  | Organisations scoring highly on Friends and Family/Safety thermometer and CQC data will be given priority over others that are equally ranked |
| We will give priority to sites which plan to deliver the training to another group of staff during the life-time of our evaluative study, as this will allow a pre-post evaluation of staff knowledge, attitudes and values. | We will approach the top ranked sites to explain case study participation and ask if each site might be willing to take part. If so, we will ask if the site is planning to deliver training to a group of staff during the life-time of the evaluation.  As we approach successive sites, we will consider the range we are finding in terms of format of training, area served (urban or rural, region) and configuration of services. We will use purposive sampling as necessary to achieve variety within the ranked groupings. |
| We will aim for variation, if possible, in respect of format of training, and context (population, ethnic diversity, and configuration of services) as this will allow a variety of contexts to be explored and so allow the findings of the study to have broader relevance. |  |
